# A Dioxobilin-Type Fluorescent Chlorophyll Catabolite as a Transient Early Intermediate of the Dioxobilin-Branch of Chlorophyll Breakdown in *Arabidopsis thaliana*

**DOI:** 10.1002/anie.201506299

**Published:** 2015-10-01

**Authors:** Iris Süssenbacher, Stefan Hörtensteiner, Bernhard Kräutler

**Affiliations:** Institut für Organische Chemie und Centrum für Molekulare Biowissenschaften Universität Innsbruck, Innrain 80/82, 6020 Innsbruck (Austria); Institut für Pflanzenbiologie Universität Zürich, Zollikerstrasse 107, 8008 Zürich (Switzerland)

**Keywords:** chlorophylls, isomerization, phyllobilin, structure elucidation, tetrapyrrole

## Abstract

Chlorophyll breakdown in higher plants occurs by the so called “PaO/phyllobilin” path. It generates two major types of phyllobilins, the characteristic 1-formyl-19-oxobilins and the more recently discovered 1,19-dioxobilins. The hypothetical branching point at which the original 1-formyl-19-oxobilins are transformed into 1,19-dioxobilins is still elusive. Here, we clarify this hypothetical crucial transition on the basis of the identification of the first natural 1,19-dioxobilin-type fluorescent chlorophyll catabolite (DFCC). This transient chlorophyll breakdown intermediate was isolated from leaf extracts of *Arabidopsis thaliana* at an early stage of senescence. The fleetingly existent DFCC was then shown to represent the direct precursor of the major nonfluorescent 1,19-dioxobilin that accumulated in fully senescent leaves.

About 25 years ago, when chlorophyll (Chl) seemed to disappear in plants without leaving a trace,^1^ a nonfluorescent Chl catabolite (NCC) was identified as a 1-formyl-19-oxobilin-type linear tetrapyrrole,^2^ thereby opening the door to the structure-guided discovery of the “PaO/phyllobilin” pathway of Chl breakdown.^3^ Oxidative cleavage of the Chl macroring generates 1-formyl-19-oxobilins and sets the stage for the formation of various bilin-type catabolites of Chl, the “phyllobilins”.3b,d In addition, as was recognized recently, Chl breakdown “branches out”, and furnishes 1,19-dioxobilin-type chlorophyll catabolites (DCCs) as a second major family of phyllobilins.3c,d The latter (“type-II”) phyllobilins are mostly colorless, such as the 1,19-dioxobilin-type NCCs (DNCCs).^5^ Originally, DNCCs were suggested to be deformylation products of NCCs,5a and the step by which 1-formyl-19-oxobilins are converted into DCCs remained to be identified.3d Indeed, the puzzling stereochemical diversity displayed by natural DNCCs indicated NCCs as unlikely precursors of DNCCs.5b We thus suggested an earlier branching point in the “PaO/phyllobilin” pathway of Chl breakdown that involved an unknown dioxobilin-type fluorescent Chl catabolite (DFCC) as the precursor of DNCCs (Figure 1).5b

**Figure 1:**
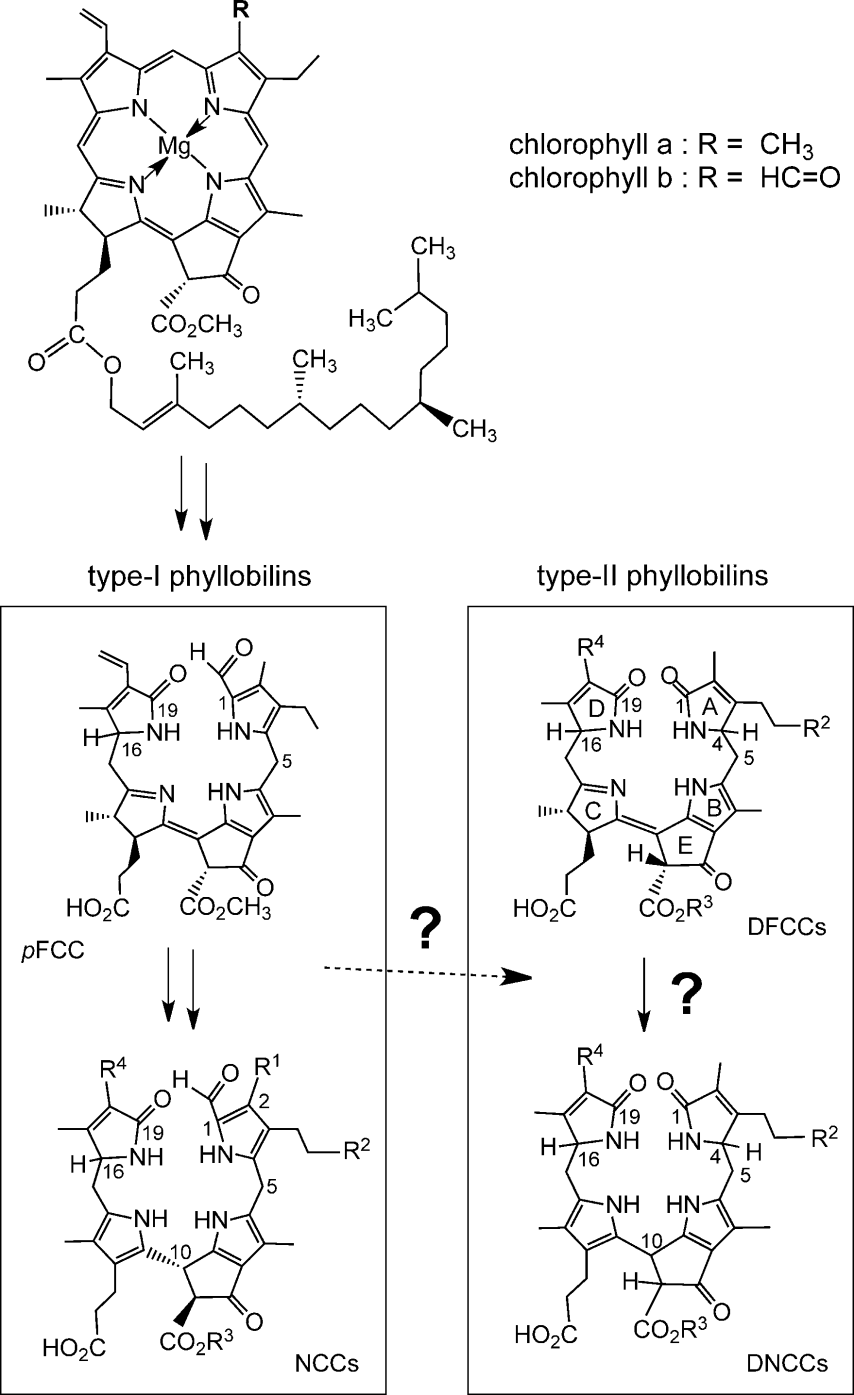
Abridged structural outline of Chl breakdown in higher plants, in which the two major branches of phyllobilins are highlighted. Type-I phyllobilins (1-formyl-19-oxobilins) are formed first (left), while those of type-II are generated subsequently (1,19-dioxobilins, right). The natural branching point(s) from type-I to type-II phyllobilins remain(s) to be identified.

A remarkable cytochrome P450 enzyme (CYP89A9) was identified recently in *Arabidopsis thaliana (A. thaliana)* that catalyzed the in vitro deformylation of the “primary” fluorescent Chl catabolite (*p*FCC) to the corresponding epimeric “primary” DFCCs (*p*DFCCs).^6^ In weakly acidic solution, such a pair of *p*DFCC epimers isomerized rapidly to a pair of DNCCs. Thus, two key steps of the “dioxobilin” branch of chlorophyll breakdown appeared to be clarified.^6^ Unfortunately, these results provided no conclusion with respect to the stereochemical outcome of the hypothetical DFCC-DNCC isomerization,5b nor was a major natural step of the dioxobilin path clearly identified by the in vitro enzyme reaction. We have now “trapped” a transiently existent, natural DFCC in an early senescence stage of de-greening leaves of the model plant *A. thaliana*, and have explored its isomerization to a DNCC. This isomerization occurred rapidly, was highly stereoselective, and cleanly furnished *At*-DNCC-33, which is the major natural DNCC in senescent leaves of *A. thaliana* (this DNCC was provisionally named *At*-NDCC-1 previously).^6^

To trap early intermediates of Chl breakdown in wild-type *A. thaliana*, fresh leaf extracts were analyzed after two days of incubation in the dark, that is, at an early stage of senescence. Analysis by HPLC revealed a variety of Chl catabolites (see Figure 2 and Figure S2 in the Supporting Information), consistent with similar earlier observations.^6^, ^7^ Nonfluorescent fractions were observed with absorptions near 315 nm (classified as NCCs), as well as more prominent compounds with weak absorptions near 237 nm and 274 nm, but none near 315 nm, and thus were provisionally classified as NDCCs (“nonfluorescent” DCCs). Strikingly, a minor fluorescent fraction was also detected that showed two characteristic bands near 237 nm and near 360 nm (see Figure 3), which were conspicuously similar to those of DFCCs characterized earlier as the product of the P450-catalyzed deformylation of *p*FCC.^6^

**Figure 2:**
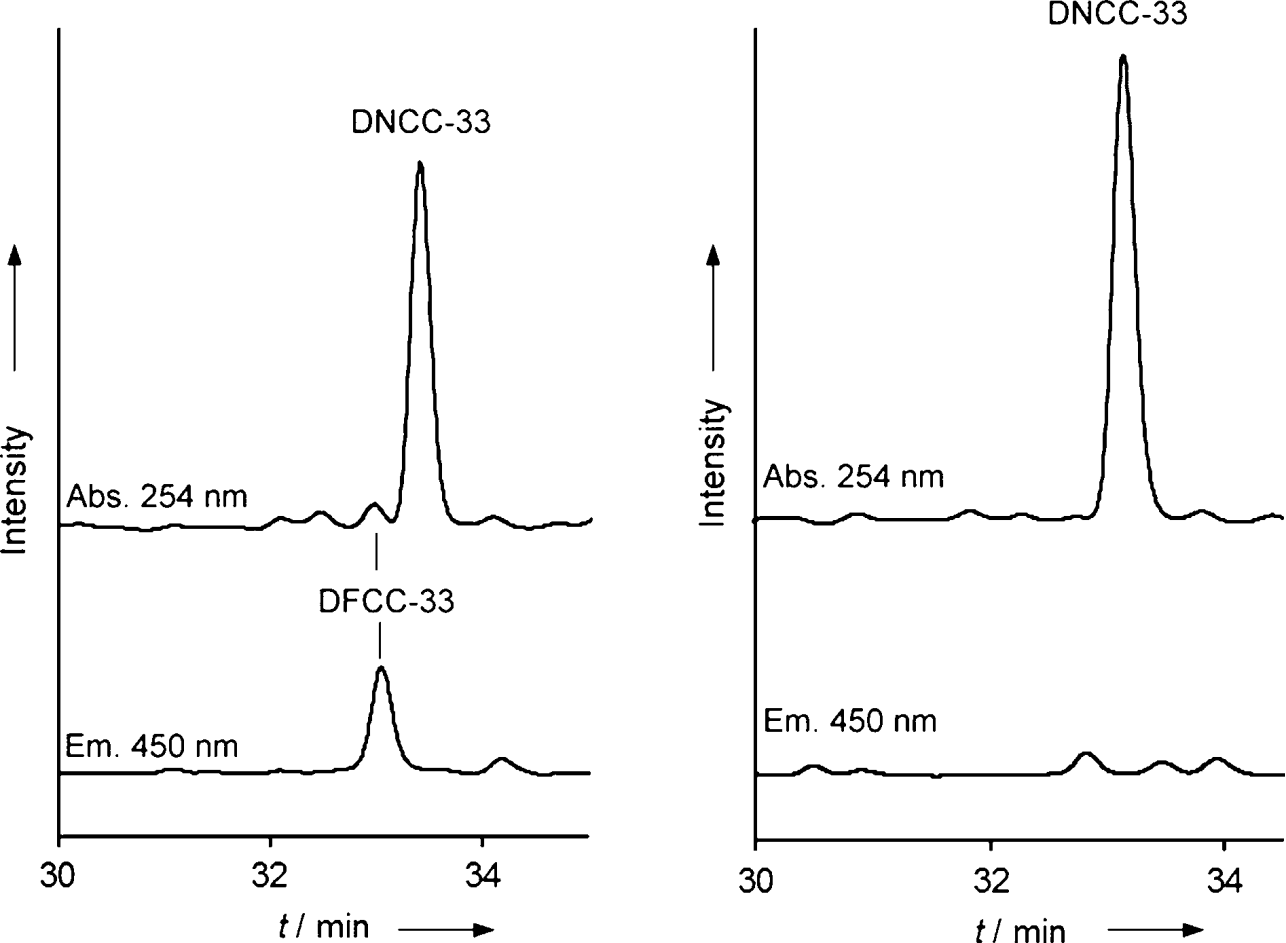
HPLC analysis of chlorophyll catabolites in leaf extracts of *A. thaliana* (wild-type). Left: Analysis at an early stage of senescence (leaves kept in the dark for 2 days); Right: Analysis of de-greened leaves kept in the dark for 6 days (top traces: absorbance at 254 nm, bottom traces: luminescence at 450 nm; see Experimental Section and Supporting Information for details).

**Figure 3:**
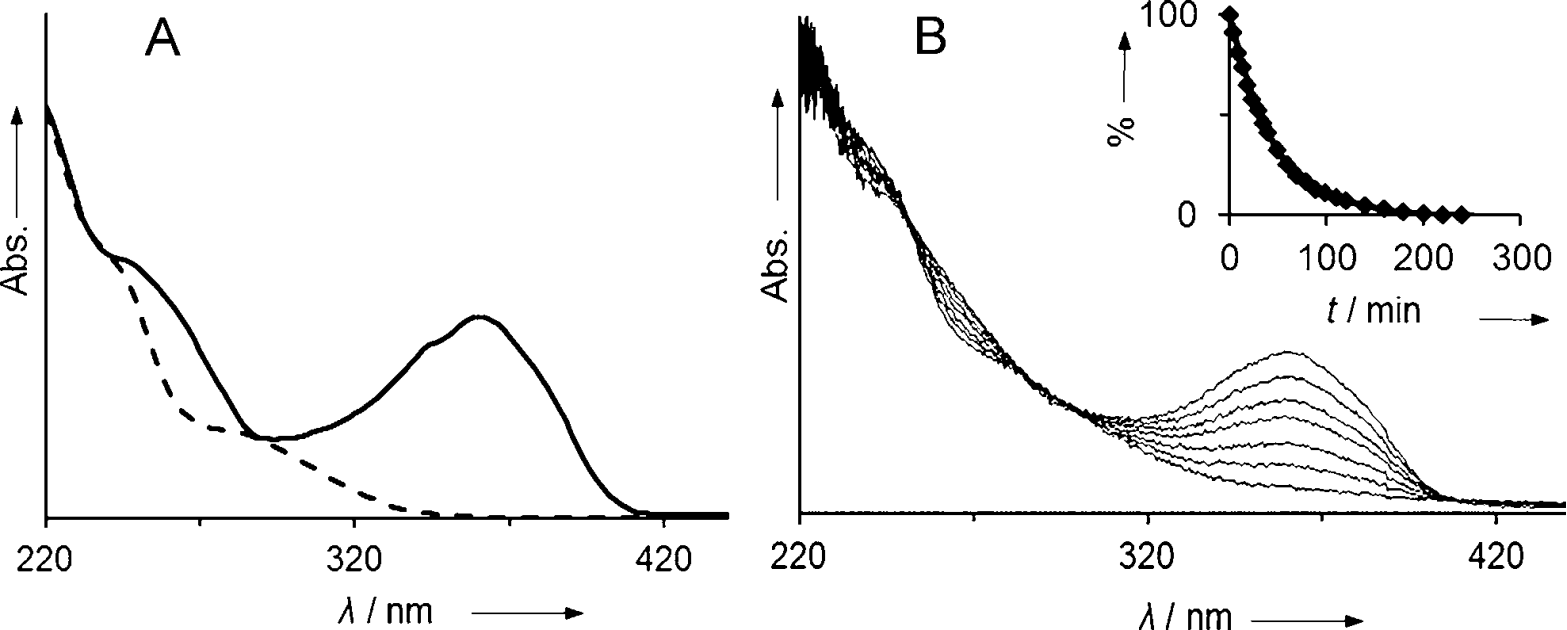
A) UV spectra *of At*-DFCC-33 (1, solid line) and *At*-DNCC-33 (2, dashed line). B) UV/Vis analysis of the isomerization of *At*-DFCC-33 (1) to *At*-DNCC-33 (2), observed in MeOH/aq potassium phosphate (100 mm, pH 5)=1:1.4; traces recorded at times 1, 10, 20, 30, 50, 80, and 220 min. Inset: Absorbance changes at 360 nm follow first order kinetics (*k*=0.022 min^−1^).

Roughly 130 μg of the unknown fluorescent compound **1** with a standard retention time of 32.8 min was isolated by semipreparative HPLC from *A. thaliana* leaves, kept in the dark for two days (212 g) or three days (148 g). Analysis of **1**, now named *At*-DFCC-33, by positive-ion ESI mass spectrometry, revealed a pseudo-molecular ion [*M*+H]^+^ at *m*/*z* 619.0, consistent with a molecular formula of C_33_H_38_N_4_O_8_. Fragments at *m*/*z* 575.1 and 434.2 indicated subsequent loss of CO_2_ and of ring A. Thus, the DFCC **1** was revealed to be an isomer of *At*-DNCC-33 (**2**), the main Chl catabolite in senescent leaves of *A. thaliana* (previously *At*-NDCC-1).^6^, ^8^

Signals of 31 (of its 32) carbon-bound hydrogen atoms were observed and assigned in a 600 MHz ^1^H NMR spectrum of *At*-DFCC-33 (in CD_3_OD, 273 K). Among them were three methyl group singlets (at high field) and a doublet at *δ*=1.15 ppm, assigned to the H_3_C13^1^ methyl group on the basis of correlations in 2D NMR spectra. A multiplet at *δ*=2.79 was assigned to HC13, and another, at *δ*=2.74, to the direct neighbor HC12. From analysis of the set of correlations from ^1^H,^1^H-ROESY, ^1^H,^1^H-COSY, ^1^H,^13^C-HSQC, and ^1^H,^13^C-HMBC spectra, the constitution of rings B and ring C was deduced to be the same as in FCCs.^9^ Two dd at *δ*=4.43 ppm and *δ*=4.83 ppm indicated hydrogen atoms at positions C4 and C16, as observed earlier in spectra of DNCCs.^5^, ^6^ In addition, a 2-hydroxyethyl side chain was identified at position C3. Analysis of the 2D NMR spectra revealed the structure of *At-*DFCC-33 (**1**, see Figure S6 in the Supporting Information). The derived structure is consistent with the observed UV spectrum, in which an absorption band near 360 nm is seen, characteristic of the common B/C chromophore of FCCs and DFCCs. An additional absorption band near 320 nm was absent, which is a characteristic of the formylpyrrole unit of the type-I phyllobilins, such as FCCs and NCCs. Thus, a first representative of the elusive natural DFCCs could be characterized.

*At*-DFCC-33 (**1**) exhibited the expected instability at room temperature, when dissolved in unbuffered or slightly acidic aqueous media. Indeed, as known from previous studies, typical FCCs exist only fleetingly in weakly acidic aqueous solution, as found in vacuoles, and isomerize stereoselectively to the corresponding NCCs.^10^ Likewise, at pH 5, a sample of DFCCs from in vitro CYP89A9-deformylation of *p*FCC isomerized to a pair of DNCCs.^6^ To characterize the isomerization of the isolated, native *At*-DFCC-33 (**1**), and to identify its isomerization product, DFCC **1** was stored in potassium phosphate buffer (100 mm) at pH 5 at room temperature. A highly stereoselective conversion of the DFCC **1** was observed, and monitored by UV/Vis spectroscopy. Under these conditions, **1** exhibited a half-life of 32 min and isomerized with first order kinetics (*k*=0.022 min^−1^). After 220 min, the solution exhibited the typical UV spectrum of a DNCC (see Figure 3 B). A single product was formed (HPLC), which had the retention time of authentic *At*-DNCC-33 (**2**, see Figure S8 in the Supporting Information). A positive-ion ESI mass spectrum of the presumed isomerization product was also consistent with the molecular formula C_33_H_38_N_4_O_8_ ([*M*+H]^+^ at *m*/*z* 619.0). A CD spectrum of the isomerization product of the DFCC **1** showed the same features as authentic *At-*DNCC-33 (**2**), and of other DNCCs isolated from senescent leaves of *A. thaliana* (see Figure S1 in the Supporting Information).^8^ The nonfluorescent product of the acid-induced isomerization of *At*-DFCC-33 (**1**) was, thus, identified as *At*-DNCC-33 (**2**), the main Chl catabolite found in senescent leaves of *A. thaliana*.^6^, ^8^

The discovery of a natural 1,19-dioxobilin-type FCC (DFCC), as well as its selective isomerization to the DNCC **2**, reported here, support the crucial role of such fleetingly existent fluorescent intermediates of Chl breakdown in a higher plant, such as (wild-type) *A. thaliana*. Interestingly, in recent work on Chl catabolites of senescent leaves of an *A. thaliana* mutant, a modified “fluorescent” 1,19-dioxobilin-type Chl catabolite (an FDCC) was identified, which carried a puzzling “extra” hydroxymethyl group, a feature also found in some NDCCs from the mutant plant,^11^ as well as in wild-type *A. thaliana.*^8^ *At*-DFCC-33 (**1**) was obtained here as a single stereoisomer, and an epimer of **1** was not identified in the leaf extract. Based on this finding, the hypothetical stereoselective formation of **1** is indicated to take place through enzymatic, oxidative in vivo deformylation of an FCC precursor. The “puzzling” hydroxymethylations that accompany the in vivo,^8^, ^11^ but not the in vitro,^6^ deformylation of *p*FCC, are not features of the natural pathway to DFCC **1**.

In this latter respect, the presence of a primary hydroxy group in *At*-DFCC-33 (**1**) at the side chain extending from C3 appears to be a crucial element,^8^ thereby suggesting a corresponding hydroxylated FCC as precursor. Indeed, hydroxylation of *p*FCC at the C3 side chain may take place in the chloroplast,3b thereby furnishing the known 3^2^-OH-*p*FCC.10c After its exit from this organelle, this FCC would be an excellent substrate for CYP89A9 (Figure 4).^6^ Enzyme-catalyzed deformylation of 3^2^-OH-*p*FCC10c leads to 3^2^-OH-*p*DFCC (**3**).^6^ Hydrolysis of the methyl ester group of **3** by MES16, the highly active methyl esterase in wild-type *A. thaliana*,10c would generate *At*-DFCC-33 (**1**), which would isomerize to the DNCC **2**, once transported into the slightly acidic vacuole (Figure 5).

**Figure 4:**
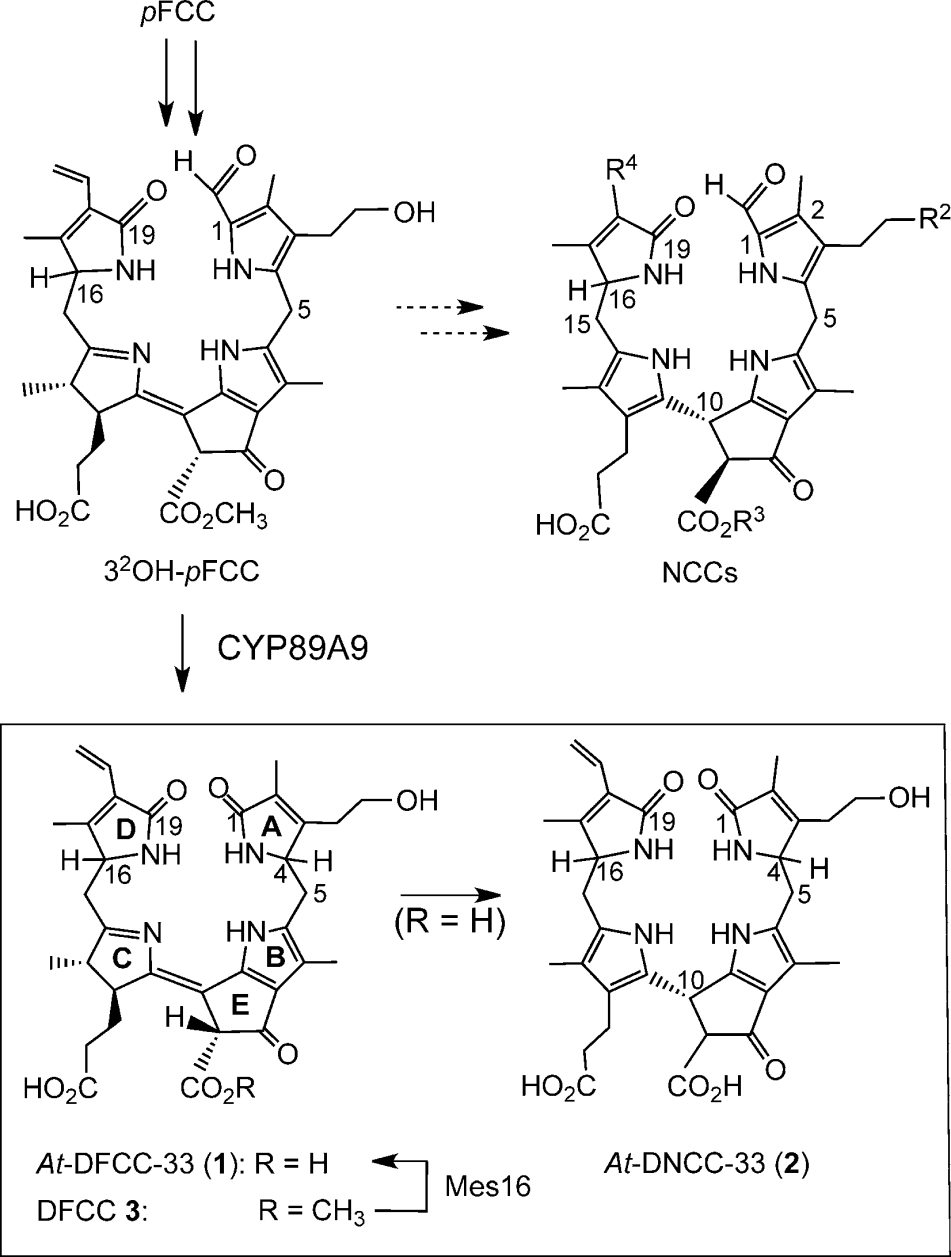
Type-I and type-II phyllobilins from the branching of Chl breakdown. 3^2^OH-*p*FCC is the “last” common precursor of NCCs (top) and of DNCC 2 (bottom). Outline of a proposed pathway of the formation of the DFCC 1 from *p*FCC by hydroxylation to 3^2^-OH-*p*FCC, followed by deformylation to the hypothetical 3^2^-OH-DFCC 3 and hydrolysis of the methyl ester group of 3. Subsequent in vivo isomerization of the DFCC 1 furnishes the DNCC 2.

**Figure 5:**
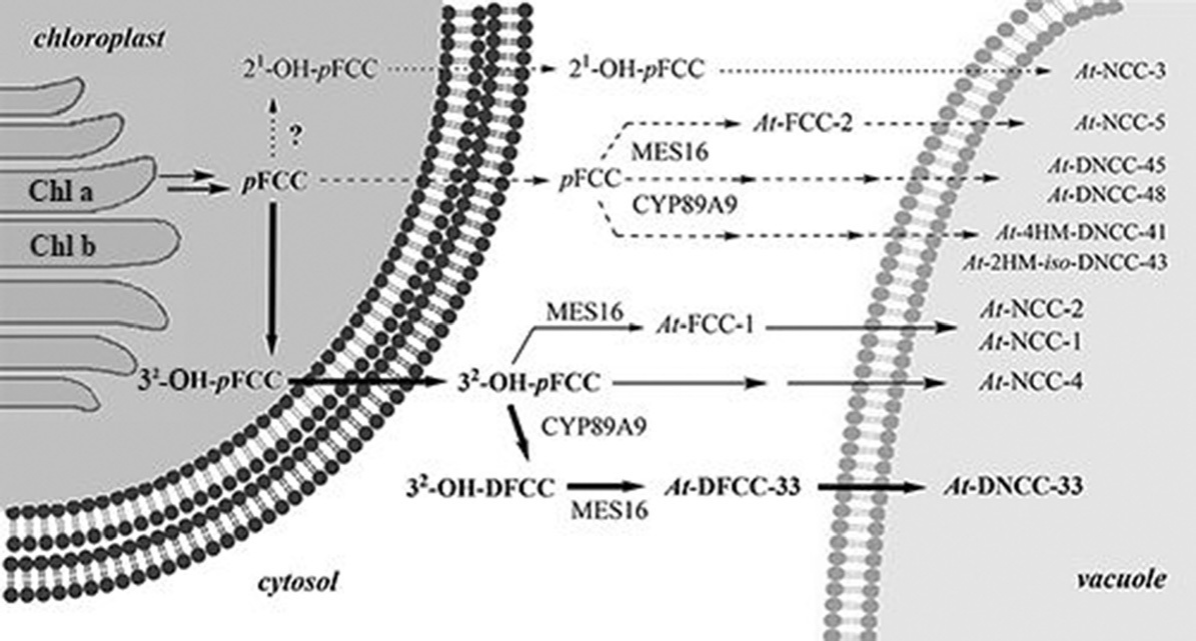
Topographical model of hypothetical steps of Chl breakdown in senescent *A. thaliana* leaves, highlighting the major steps with abridged names of known Chl catabolites.

Indeed, the “fate” of FCCs upon exit from the chloroplast depends primarily upon the interaction with CYP89A9 and/or MES16—two “competing” cytosolic enzymes. MES16 transforms FCCs into polar FCC-diacids, which appear to be inefficient substrates for CYP89A9. Indeed, O8^4^-desmethyl-*p*FCC (*At*-FCC-2,7b see Figure S1 in the Supporting Information) was not (in vitro) deformylated by CYP89A9.^6^ Thus, formation of DFCC **1** from 3^2^-OH-*p*FCC may not occur through hydrolysis of 3^2^-OH-*p*FCC by MES16 to give 3^2^-OH-O8^4^-demethyl-*p*FCC (also known as *At-*FCC-1),7b followed by deformylation of *At*-FCC-1 by CYP89A9 to **1**. Instead, two NCCs are found in *A. thaliana* leaves that are suggested to be downstream products from intact *At*-FCC-1.7b

Under standard conditions of extract preparation and isolation,^11^ *At*-DFCC-33 (**1**) could not be isolated in pure form, and solutions of **1** had to be kept cold (0 °C or less) to minimize isomerization of **1** to **2**. Rapid conversion of the DFCC **1** into a DNCC was not unexpected, considering the known isomerization of FCCs to NCCs. Such isomerizations were observed to be particularly fast in FCCs with a free 8^2^-carboxylic acid function,10c which is also present in the DFCC **1**. As originally delineated for “primary” FCCs,10a,b the propionic acid function at C12 has been proposed to induce the steroselective isomerization of **1** to **2**, which, therefore, would be deduced to generate **2** with *R* configuration of the asymmetric methine group at C10. Indeed, the isomerization of **1** at pH 5 produced the natural DNCC **2**, whose chiroptical features fall in line with those of most DNCCs identified previously.5a, 6, 11 Interestingly, this stereochemical outcome is contrary to the stereochemistry of the apparently “aberrant” case of the DNCC from Norway maple,5b which, thus, still requires an alternative explanation.

With the advent of the characterization of *At*-DFCC-33 (**1**), an early “bona fide’ intermediate of the major dioxobilin-branch of Chl breakdown in *A. thaliana* is now identified. The DFCC **1** is generated as a transient intermediate near the hypothetical branching point of the PaO/phyllobilin pathway at which the type-II phyllobilins diverge from the first formed 1-formyl-19-oxobilin-type Chl catabolites (or type-I phyllobilins). Identification of **1** corroborates the hypothetical role of DFCCs as natural, short-lived entry points to nonfluorescent type-II phyllobilins, such as the abundant DNCCs. The fleeting existence of *At*-DFCC-33 (**1**) also made it necessary to isolate **1** from leaves at an early stage of senescence. In fully senescent, yellow *A. thaliana* leaves, fluorescent phyllobilins are hardly detectable. However, a range of colorless Chl catabolites (NCCs, DNCCs, and NDCCs) were identified as products further downstream of the two breakdown branches.^6^–^8^

As deduced for the natural formation of NCCs from the corresponding FCCs,10a,b a slightly acidic medium, as provided in the vacuoles, is beneficial for the rapid isomerization of DFCCs to the corresponding DNCCs. Indeed, at pH 5 DFCC **1** undergoes rapid stereoselective isomerization to DNCC **2**, thus suggesting DFCC **1** is the natural precursor of **2** in *A. thaliana*. Since DNCC **2** represents, by far,^6^ the major fraction among the phyllobilins in senescent leaves of this plant (see Figure 2 and Figure S1 in the Supporting Information), this, in turn, gives the transient DFCC **1** an important position in Chl breakdown in such senescent leaves. According to the model of Chl breakdown in higher plants,3c,d import of the DFCC **1** into the vacuoles would be required to set the stage for the isomerization to the DNCC **2**.

The critical in vivo transition from 1-formyl-19-oxobilin-type phyllobilins to 1,19-dioxobilin-type (or type-II) phyllobilins would, thus, mostly occur by deformylation of 3^2^-OH-*p*FCC, an excellent in vitro substrate for the FCC-deformylase CYP89A9 (Figure 5).^6^ The deformylation product 3^2^-OH-*p*DFCC (**3**), from which the DFCC **1** is presumably generated by the cytosolic methyl esterase MES16 has, so far, remained unidentified in *A. thaliana* leaf extracts. Deformylation of the original *p*FCC also occurs on a minor additional path in senescent *A. thaliana* leaves.^8^ It is deduced to give rise to elusive *p*DFCCs, and to “puzzling” hydroxymethylated *iso*-DFCCs as precursors of the corresponding group of remarkable NDCCs, which were only recently identified.^8^, ^11^ Hence, branching of the PaO/phyllobilin path towards type-II phyllobilins occurs in more than one case subsequent to formation of the colorless *p*FCC. Two critical branching points from type-I to type-II phyllobilins have now been identified, consistent with the known low (in vitro) selectivity of the deformylase CYP89A9.^6^ Hence, the findings reported here allow a deep glimpse into Chl breakdown in *A. thaliana*, which, while adhering to the PaO/phyllobilin pathway, takes divergent roads at later stages (Figure 5).

Breakdown of Chl in senescent *A. thaliana* leaves shows hallmarks of a “detoxification” process:3a it rapidly leads to a variety of increasingly polar, colorless, and nonfluorescent catabolites, among which type-II phyllobilins dominate, such as the DNCC **2**. Their intriguing 1,19-dioxobilin-type structure is a constitutional feature shared with the heme-derived bilins.^12^ This common structural property of type-II phyllobilins, and of the heme-derived “bile pigments”, is remarkable in light of the diverse important biological roles of heme-derived bilins.^12^ Hence, in view of their unique chemistry, the ubiquitous phyllobilins3d, 4 also qualify as candidates for relevant physiological roles in higher plants, as well as, probably, in plant-eating animals and humans.^13^ However, a physiological effect of phyllobilins remains remarkably elusive.

## Experimental Section

HPLC analysis: 207 mg of fresh *A. thaliana* (wild-type) leaves, kept in the dark for 2 days, were ground under N_2_, extracted with 41 μL MeOH, and centrifugated for 10 min. The clear supernatant (160 μL) was diluted 1:1 with potassium phosphate buffer (pH 7). After further centrifugation at 13 000 rpm for 2 min, a 20 μL aliquot was analyzed by HPLC (standard conditions; for details see the Supporting Information).

Characterization of *At-*DFCC-33 (**1**): UV/Vis (Hitachi U-3000, MeOH, *c*=ca. 3.5×10^−5^ m). *λ*_max_ (*ε*_rel_)=360 (1.00), 244 sh (1.63); CD (Jasco 715, MeOH, *c*=ca. 3.5×10^−5^ m). *λ*_max/min_ [nm] (Δ*ε*_rel_)=357 (2.5), 285 (−0.37), 264 (1.4), 240 (−1.6), 224 (0.21). ^1^H and ^13^C NMR (600 MHz; CD_3_OD, 275 K): see the Supporting Information. LC-ESIMS: *m*/*z* (%): 657.0 (4, [*M*+K]^+^); 621.0 (9), 620.0 (37), 619.0 (100, C_33_H_39_N_4_O_8_, [*M*+H]^+^); 613.1 (8, [*M*-CO_2_+K]^+^); 601.1 (8, [*M*-H_2_O+H]^+^); 597.2 (9, [*M*-CO_2_+Na]^+^); 577.1 (9), 576.1 (36), 575.1 (98, [*M*-CO_2_+H]^+^); 434.2 (60, [*M*-CO_2_-C_7_H_11_NO_2_ (ring A)+H]^+^; see the Supporting Information for further details).

For details of the isolation of **1**, the isomerization of **1** at pH 5 (Figure 3), the characterization of *At*-DNCC-33 (**2**), formed by isomerization of **1**, as well as spectroscopic details see the Supporting Information.

## Supporting Information

As a service to our authors and readers, this journal provides supporting information supplied by the authors. Such materials are peer reviewed and may be re-organized for online delivery, but are not copy-edited or typeset. Technical support issues arising from supporting information (other than missing files) should be addressed to the authors.

miscellaneous_information
