# Supplementary material for: A Dioxobilin-Type Fluorescent Chlorophyll Catabolite as a Transient Early Intermediate of the Dioxobilin-Branch of Chlorophyll Breakdown in Arabidopsis thaliana
Source: Angew Chem Int Ed Engl. 2015 Oct 1;54(46):13777–81. doi: 10.1002/anie.201506299 (PMC4678512; doi:10.1002/anie.201506299)
Supplement: Supplementary file 1 [file anie0054-13777-sd1.pdf]

Angewandte Chemie
Eine Zeitschrift der Gesellschaft Deutscher Chemiker

Supporting Information

A Dioxobilin-Type Fluorescent Chlorophyll Catabolite as a Transient Early Intermediate of the Dioxobilin-Branch of Chlorophyll Breakdown in *Arabidopsis thaliana*

*Iris Süssenbacher, Stefan Hörtensteiner, and Bernhard Kräutler**

anie_201506299_sm_miscellaneous_information.pdf

Materials and Methods

Plant material.

Arabidopsis thaliana ecotype Col-0 was used as the wild type. Plants were grown on soil in 8-h-light/16-h-dark photoperiod under fluorescent light of 80 to 120 µmol photons m^{-2} s^{-1} at 22°C and 60% relative humidity. For senescence induction, leaves from 8-week-old plants were excised and incubated in permanent darkness on wet filter paper at ambient temperature.[1]

Chemicals.

HPLC-grade methanol (MeOH) and n-hexane were from *VWR* (Leuven, Belgium) and *Acros Organics* (Geel, Belgium). Potassium dihydrogen phosphate puriss. p.a, potassium phosphate dibasic-anhydrous puriss. p.a and ammonium acetate were from *Fluka* (Buchs, Switzerland). Ultrapure water (18 MΩcm^{-1}) was from a *Millipore* apparatus, 5.g and 1.g Sep-Pak C18 Cartridges from *Water Associates* (Milford, USA).

Chromatographic Methods

HPLC. Hewlett Packard (*hp*) series 1100 HPLC-system, online degasser (G 1322 A), *Agilent* quaternary pump (G 1311 A), diode array detector (G 1315 A) and fluorescent detector (G 1321 A) with manual sampler. Data were collected and processed with *Agilent ChemStation*.

Analytical HPLC: Injection loop 20 µL (Rheodyne valve); *Phenomenex hyperclone* ODS 5 µm 250 x 4.6 mm i.d. column (at room temperature) connected to *Phenomenex* ODS 4 x 3 mm

i.d. pre-column was used with a flow rate 0.5 ml min^{-1}. Solvent A: MeOH, solvent B: 50 mM potassium phosphate buffer (pH 7) standard solvent composition A/B: 0 – 5 min: 20/80; 5 – 55 min: 20/80 to 70/30; 55 – 60 min: 70/30 to 100/0; 60 – 70 min: 100/0; 70 – 75 min: 100/0 to 20/80. Co-injection: Solvent A: MeOH, solvent B: 50 mM potassium phosphate buffer (pH 7) standard solvent composition A/B: 0 – 2 min: 37/63; 2 – 24 min: 37/63 to 49/51; 24 – 27 min: 49/51 to 100/0; 27 – 30 min: 100/0; 30 – 33 min: 100/0 to 37/63.

Semi-preparative HPLC: Injection loop 200 µL (*Rheodyne* valve); *Phenomenex hyperclone* ODS 5 µm 250 x 4.6 mm i.d. column (at room temperature) connected to *Phenomenex* ODS 4 x 3 mm i.d. pre-column was used with a flow rate 0.5 ml min^{-1}. Re-purification of *At*-DFCC-33: A) Solvent A: MeOH, solvent B: 50 mM potassium phosphate buffer (pH 7) standard solvent composition A/B: 0 – 2 min: 32/68; 2 – 32 min: 32/68 to 39.5/60.5; 32 – 35 min: 39.5/60.5 to 43/57; 35 – 38 min: 43/57 – 100/0; 38 – 41 min: 100/0; 41 – 44 min: 100/0 to 32/68. B) Solvent A: MeOH, solvent B: 10 mM ammonium acetate (pH 7) standard solvent composition A/B: 0 – 2 min: 28/72; 2 – 32 min: 28/72 to 40/60; 32 – 35 min: 40/60 to 43/57; 35 – 38 min: 43/57 – 100/0; 38 – 41 min: 100/0; 41 – 44 min: 100/0 to 28/72.

Preparative HPLC. Injection loop 2 ml; *Phenomenex hyperclone* ODS 5 µm 250 x 21.2 mm i.d. column (at room temperature) protected with a *Phenomenex* ODS 10 x 5 mm pre-column was used with a flow rate 5 ml min^{-1}. Solvent A: MeOH, solvent B: 50 mM potassium phosphate buffer (pH 7); solvent composition A/B: 0 – 5 min: 18/82; 5 – 95 min: 18/82 to 38/62; 95 – 125 min: 38/62 to 50/50; 125 – 140 min: 50/50 to 100/0; 140 – 155 min: 100/0; 155 – 170 min: 100/0 to 18/82.

LC/ESI-MS. LC Packings UltiMate, He degasser, UVD 340U diode array detector and a Rheodyne injection valve with 30 µL loop, *Phenomenex hyperclone* ODS 5 µm 250 x 4.6 mm i.d. column (at room temperature) connected to *Phenomenex* ODS 4 x 3 mm i.d. pre-column was used with a flow rate 0.5 ml min^{-1}. Solvent A: MeOH (4 mM NH_4OAc), solvent B: 4 mM ammonium acetate buffer (pH 7) standard solvent composition A/B: 0 – 5 min: 20/80; 5 – 55 min: 20/80 to 60/40; 55 – 60 min: 60/40 to 100/0; 60 – 70 min: 100/0; 70 – 75 min: 100/0 to 20/80. Coupled with Finnigan LCQ Classic spectrometer (conditions see below).

Spectroscopic Analysis of Chl-catabolites

General: *Ultraviolet/visible (UV/Vis)*: *Hitachi U-3000* spectrophotometer, in MeOH or MeOH/potassium phosphate puffer (100 mM, pH 5); λ_{max} [nm](ε_{rel}). *Circular dichroism (CD)*: *JASCO J715*, in MeOH or MeOH/potassium phosphate puffer (100 mM, pH 5); $\lambda_{min/max}$ [nm] ($\Delta\varepsilon_{rel}$).

Nuclear magnetic resonance (*NMR*): *Bruker UltraShield 600 MHz Avance II+* spectrometer, ^{1}H-NMR (in CD_3OD, at 273 K for *At*-DFCC-1, δ (C^1HD_2COD) = 3.31 ppm [2], s, d, t, m = singlet, doublet, triplet, multiplet, signal assignment from ^{1}H,^{1}H-COSY and ^{1}H,^{1}H-ROESY

spectra); ^{13}C-NMR (in CD_3OD, at 273 K for *At*-DFCC-1, δ ($^{13}CD_3OD$) = 49.0 ppm [2], indirect assignment of signals from ^{1}H,^{13}C-HSCQ and ^{1}H,^{13}C-HMBC spectra).

Electrospray ionization mass spectrometry (ESI-MS) [3,4]: *Finnigan LCQ Classic*, ESI-source, positive ion mode, spray voltage 4.25 kV, m/z (% intensity, type of ion), signals of isotopomeric ions are listed for quasi molecular ions ($[M+H]^+$) only.

Production of Leaf Extract and Isolation of Chlorophyll Catabolites.

Extraction of At-*DFCC-33* ***1****. Arabidopsis thaliana* (wild type) leaves were kept in darkness for 2 and 3 days and then stored frozen. A batch of 360 g (total wet weight) of such frozen, greenish *A. thaliana* leaves was proportioned into 16 batches and ground in a mortar. Each sample of powdered plant material was mixed with sea sand and extracted with 20 ml MeOH. The obtained slurry was filtrated and the extraction was repeated with a total amount of 50 ml MeOH, 30 ml potassium phosphate buffer (50 mM, pH 7) and 10 ml H_2O. The green extract was washed 3 times with 120 mL of n-hexane, diluted with 900 ml potassium phosphate buffer (50 mM, pH 7) and filtrated. The mixture was loaded on a Sep-Pak Vac 20cc (5 g) C18 cartridge, washed with water (50 ml) and eluted with MeOH (15 ml). The solvent was removed under reduced pressure on a rotary evaporator. The crude product was dissolved in 2 ml methanol/potassium phosphate buffer (50:50 *v/v*) and centrifuged for 5 min at 13 000 rpm. The clear brown solution was injected into the preparative HPLC system. This process was repeated 15 times. The relevant raw fractions from 16 runs were collected and analyzed by HPLC. The fraction of *At*-DFCC-1 were diluted with water, concentrated on a Sep-Pak classic C18 cartridge and re-purified in several runs by analytical HPLC. The collected fractions of each catabolite were diluted with 4 volumes of water, applied to a Sep-Pak classic C18 cartridge, washed with 20 ml H_2O and eluted with 5 ml MeOH. The solvents were removed in vacuum and 130 µg of analytically pure *At*-DFCC-33 (**1**) was obtained as a whitish residue.

----

[1] B. Christ, I. Süssenbacher, S. Moser, N. Bichsel, A. Egert, T. Müller, B. Kräutler and S. Hörtensteiner, *Plant Cell* **2013**, *25*, 1868-1880.
[2] H.E. Gottlieb, V. Kotlyar and A. Nudelman, *J. Org. Chem*., **1997**, 62, 7512-7515.
[3] J.B Fenn, M. Mann, C.K. Meng, S.F. Wong and C.M. Whitehouse, *Science*, **1989**, 246, 64-71.
[4] T. Müller, S. Vergeiner and B. Kräutler, *Int. J. Mass Spectrom.* **2014**, *365-366*, 48-55

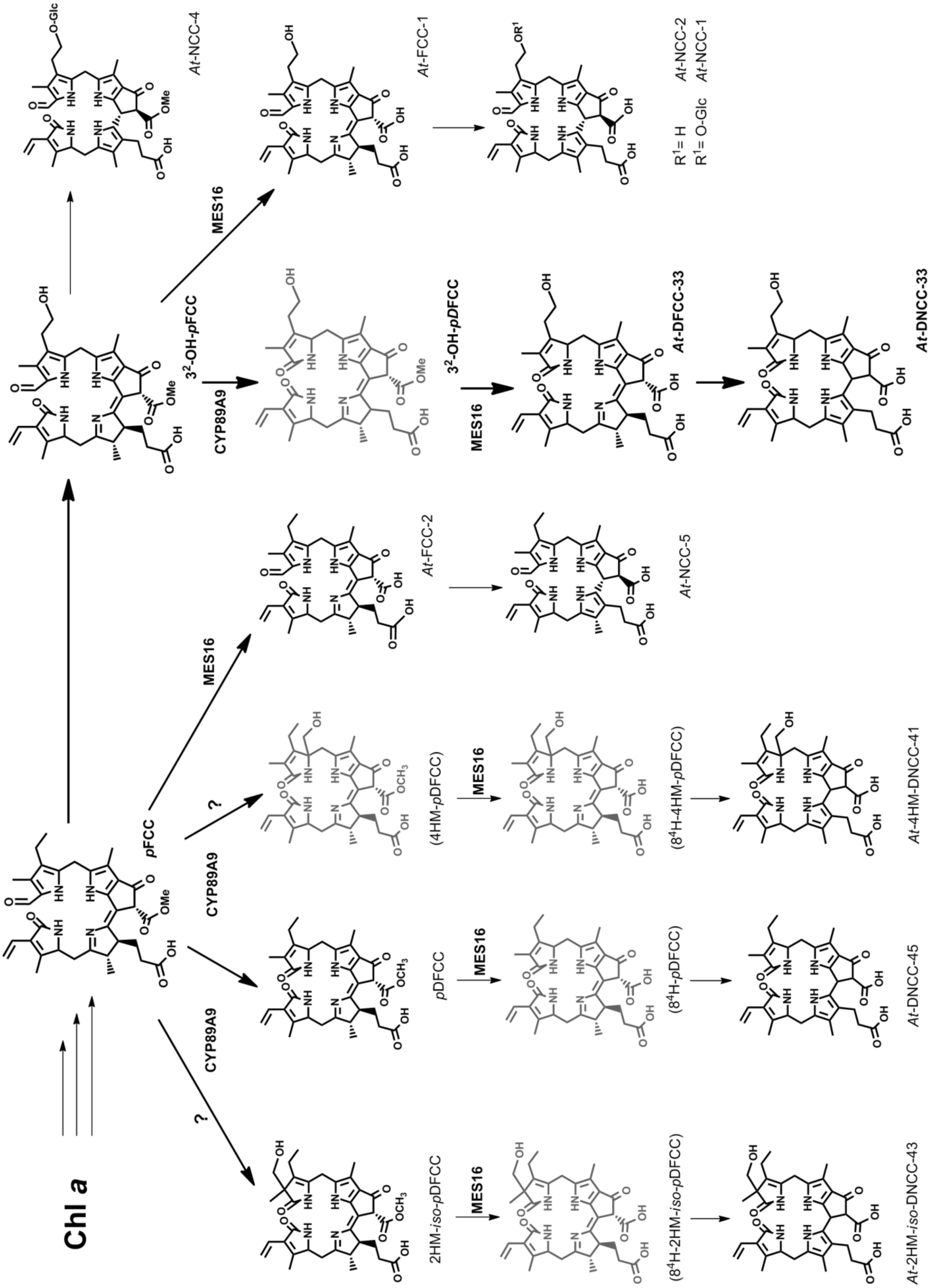

Figure S1: Hypothetical structure-based outline of Chl breakdown in *A. thaliana* leaves. Known Chl-catabolites and their abridged names of are shown in black; hypothetical intermediates are depicted in grey and their names are in brackets.

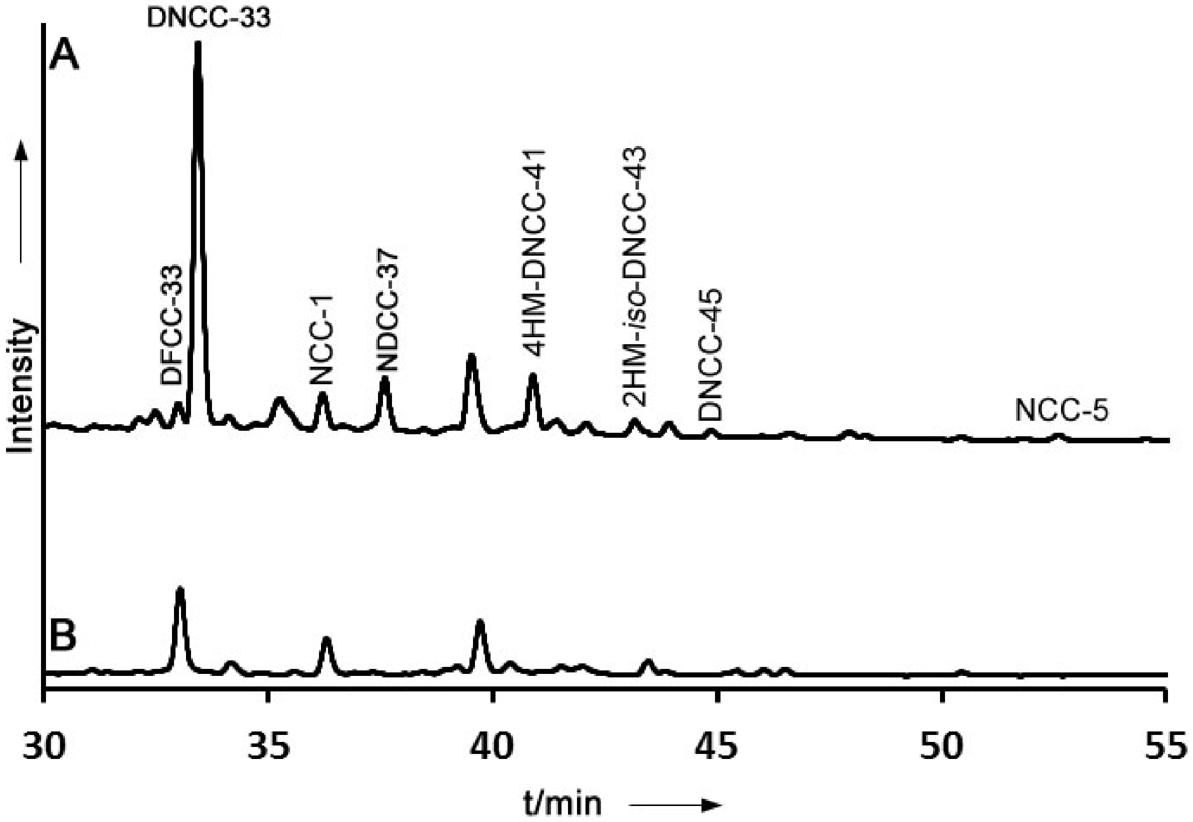

Figure S2. HPLC analysis of an extract of early senescent leaves of *A. thaliana* kept in darkness for 2 days (the now known chlorophyll catabolites in *A. thaliana* [1,5-7] are marked). A) absorbance at 254 nm, B) luminescence at 450 nm (excitation at 350 nm).

----

[5] A. Pružinska, G. Tanner, S. Aubry, I. Anders, S. Moser, T. Müller, K.-H. Ongania, B. Kräutler, J.-Y. Youn, S. J. Liljegren and S. Hörtensteiner, *Plant Physiol.* **2005**, *139*, 52-63.

[6] I. Süssenbacher, B. Christ, S. Hörtensteiner and B. Kräutler, *Chem. Eur. J.* **2014**, *20*, 87-92.

[7] I. Süssenbacher, C. Christ, S. Hörtensteiner and B. Kräutler, Chem. *Eur. J.*, DOI: 10.1002/chem.201501489.

UV/Vis and CD spectra

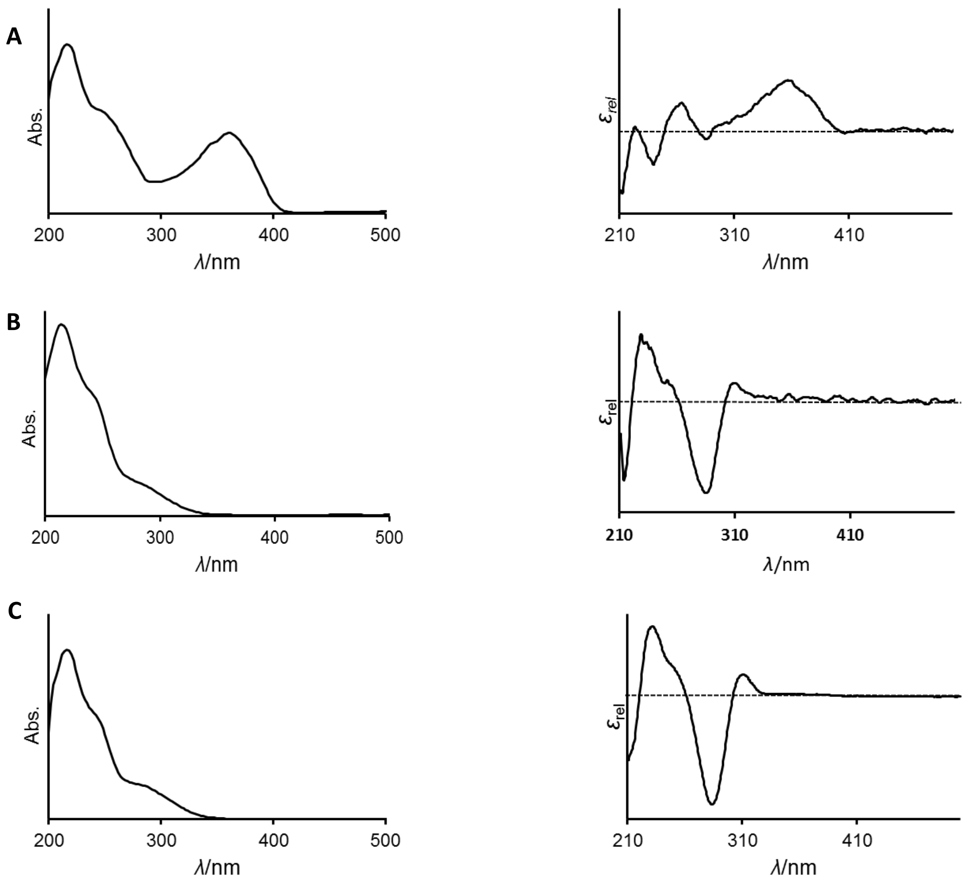

Figure S3. Online UV/Vis-spectra (left) and CD-spectra (right) A) of *At*-DFCC-33 (**1**), B) of product from isomerization of *At*-DFCC-33 (**1**), C) of authentic *At*-DNCC-33 (**2**). (Solvents: UV/Vis-spectra : A) MeOH (4mM NH_4OAc) und 4mM aqu. NH_4OAc buffer, B) and C) MeOH/potassium phosphate buffer (50 mM, pH 7)); CD-spectra: A) and C) MeOH; B) MeOH/potassium phosphate buffer (50 mM, pH 5)

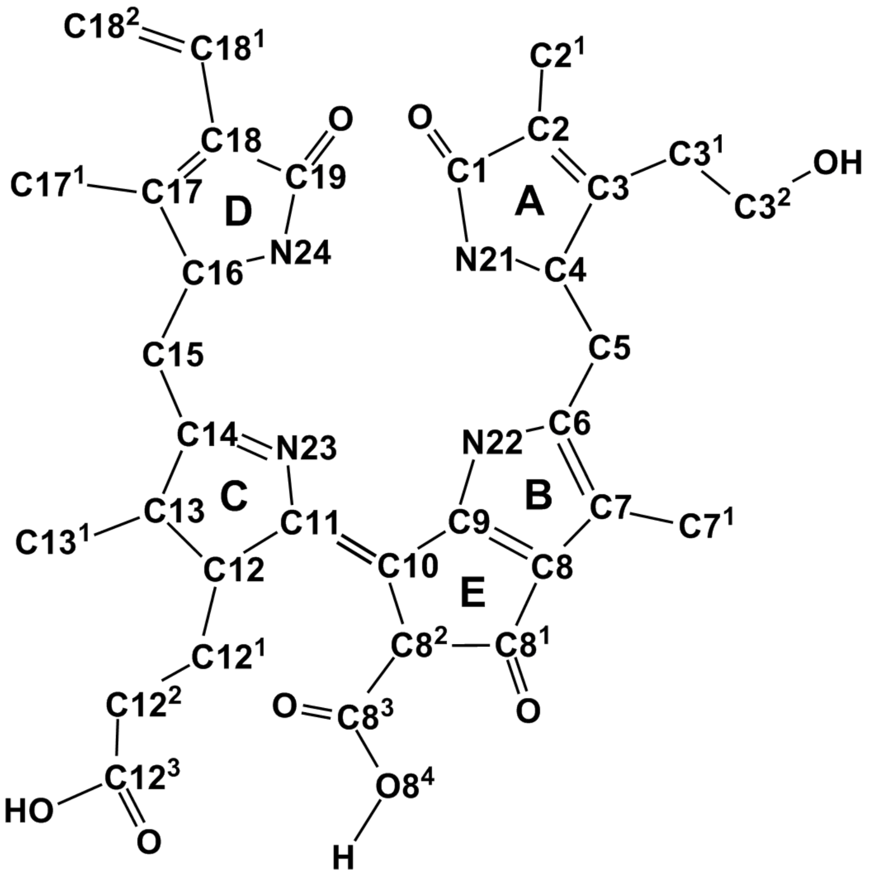

Figure S4. Atom numbering of Chl catabolites, exemplified here with the structure of *At*-DFCC-33 (**1**)

Spectral Data

At-DFCC-33 (**1**).

UV/Vis (c = ca. 3.5*10^{-5} M). λ_{max} (ε_{rel}) = 360 (1.00), 244 sh (1.63).

CD (c = ca. 3.5*10^{-5} M). $\lambda_{min/max}$ [nm] ($\Delta\varepsilon_{rel}$) = 357 (2.5), 285 (-0.37), 264 (1.4), 240 (-1.6), 224 (0.21).

^{1}H-NMR (600 MHz, CD_3OD, 273 K). δ [ppm] = 1.15 (d, *J* = 7.4 Hz, H_3C-13^1), 1.77 (s, H_3C-2^1), 2.11 (s, H_3C-7^1), 2.15 (s, H_3C-17^1), 1.86 (m, H_AC-12^1), 1.98 (m, H_BC-12^1), 2.12 (m, H_2C-12^2), 2.46 (dd, *J* = 17.5/9.7 Hz, H_AC-15), 2.51 (m, H_AC-3^1), 2.57 (dd, *J* = 14.5/9.2 Hz, H_AC-5), 2.74 (m, HC-12), 2.78 (m, H_BC-3^1), 2.79 (m, HC-13), 3.06 (dd, *J* = 17.5/3.3 Hz, H_BC-15), 3.17 (dd, *J* = 14.5/4.3 Hz, H_BC-5), 3.66 (m, H_AC-3^2), 3.70 (m, H_BC-3^2), 4.43 (dd, *J* = 4.3/9.2 Hz, HC-4), 4.83 (dd, *J* = 9.7/3.3 Hz, HC-16), 5.37 (dd, *J* = 11.7/2.3 Hz, H_AC-18^2), 6.18 (dd, *J* = 17.6/2.3 Hz, H_BC-18^2), 6.52 (dd, *J* = 17.6/11.7 Hz, HC-18^1);

^{13}C-NMR (150 MHz, CD_3OD, 273 K). δ [ppm] = 8.2 (2^1), 9.3 (7^1), 12.2 (17^1), 18.1 (13^1), 60.9 (3^2), 29.8 (5), 30.4 (12^1), 30.5 (3^1), 34.9 (15), 35.4 (12^2), 48.4 (12), 51.1 (13), 58.4 (16), 60.6 (4), 113.0 (7), 118.8 (18^2), 127.8 (8), 126.8 (18^1), 129.1 (18), 130.4 (2), 135.3 (6), 156.2 (3), 156.8 (17), 176.2 (1), 185.2 (14);

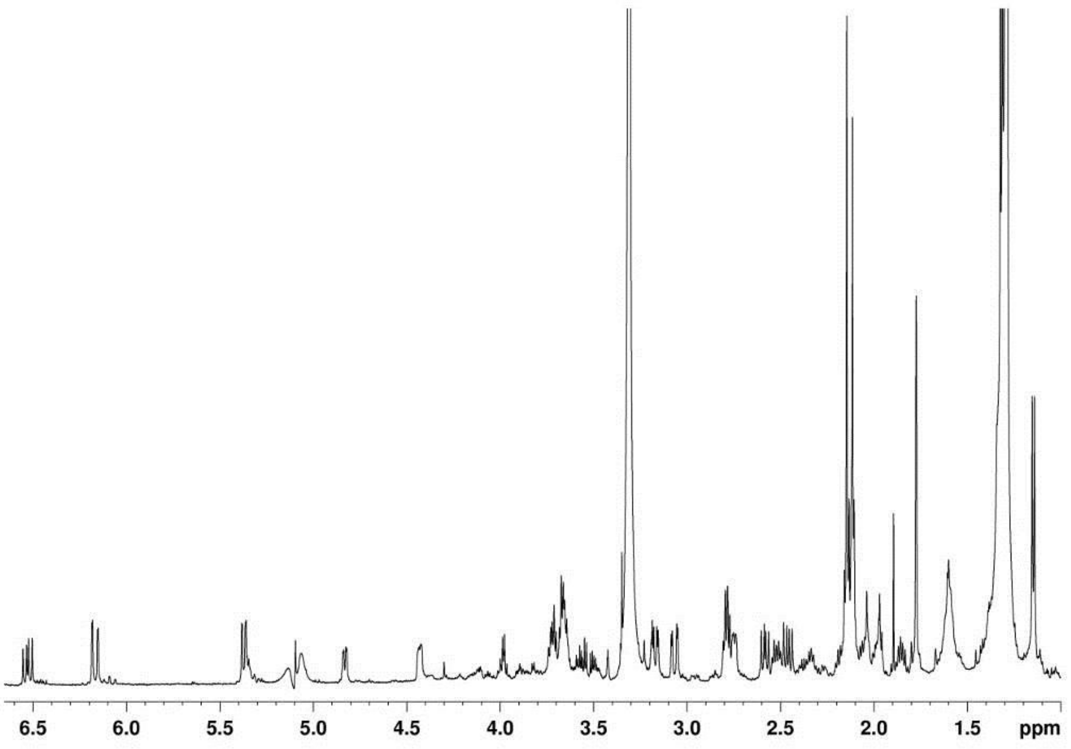

Figure S5. 600-MHz ^{1}H-NMR-spectrum of *At*-DFCC-33 (**1**) in CD_3OD at 273 K

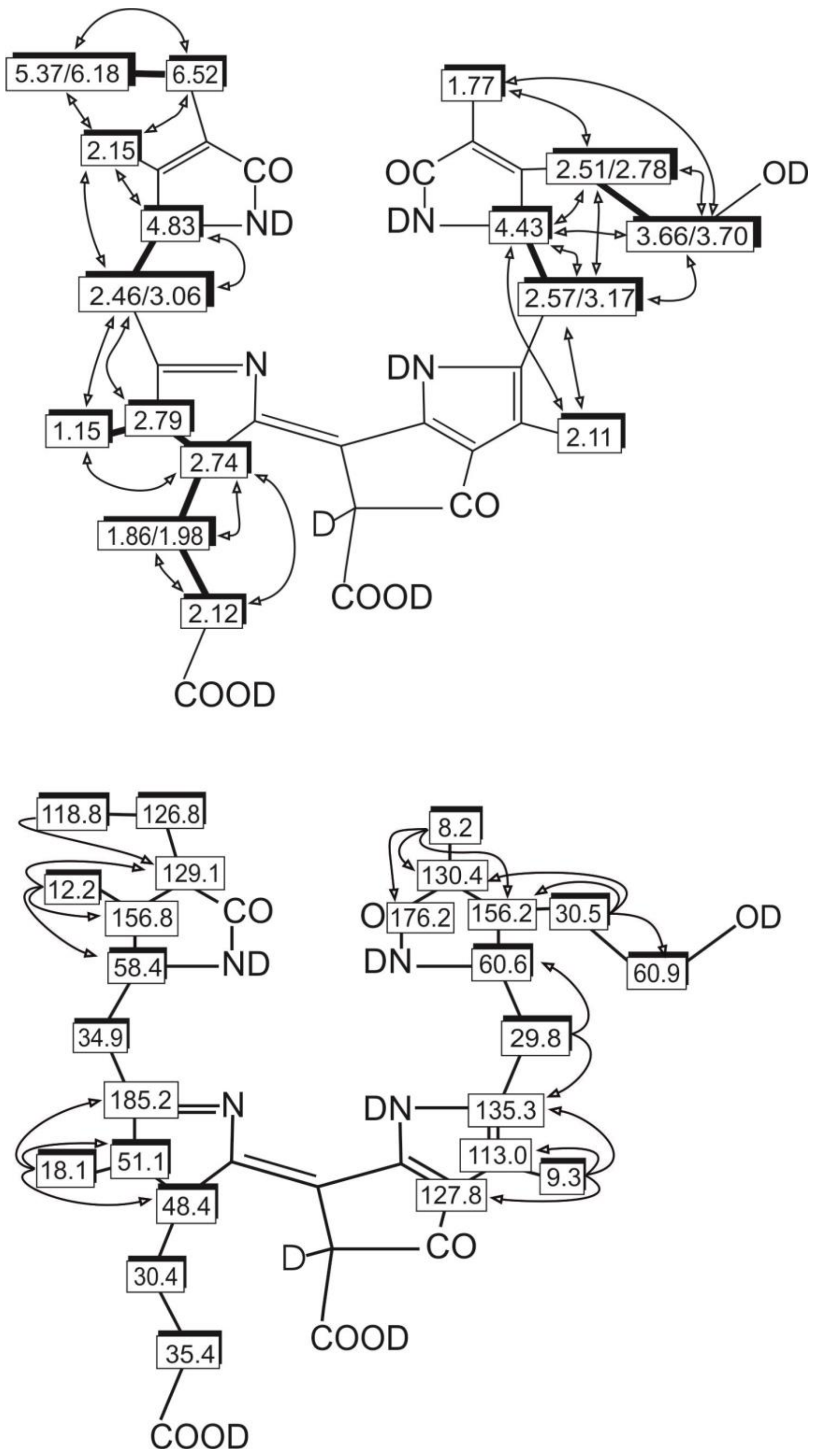

Figure S6. Graphical structural analysis of *At*-DFCC-33 (**1**) based on 600 MHz NMR-spectra (in CD_3OD, 275 K). Top: ^1H-chemical shift assignments from $^1H,^1H$-ROESY and $^1H,^1H$-COSY correlations (arrows or bold bonds, respectively). Bottom: ^{13}C chemical assignments based on direct $^1H,^{13}C$-HSQC correlations (shaded boxes) and on $^1H,^{13}C$-HMBC correlations (symbolized by arrows, open boxes).

LC-ESI-MS, m/z (%). 657.0 (4, $[M+K]^+$); 621.0 (9), 620.0 (37), 619.0 (100, $C_{33}H_{39}N_4O_8$, $[M+H]^+$); 613.1 (8, $[M-CO_2+K]^+$); 601.1 (8, $[M-H_2O+H]^+$); 597.2 (9, $[M-CO_2+Na]^+$); 577.1 (9), 576.1 (36), 575.1 (98, $[M-CO_2+H]^+$); 434.2 (60, $[M-CO_2-C_7H_{11}NO_2$ (ring A)$+H]^+$); (see Figure S7)

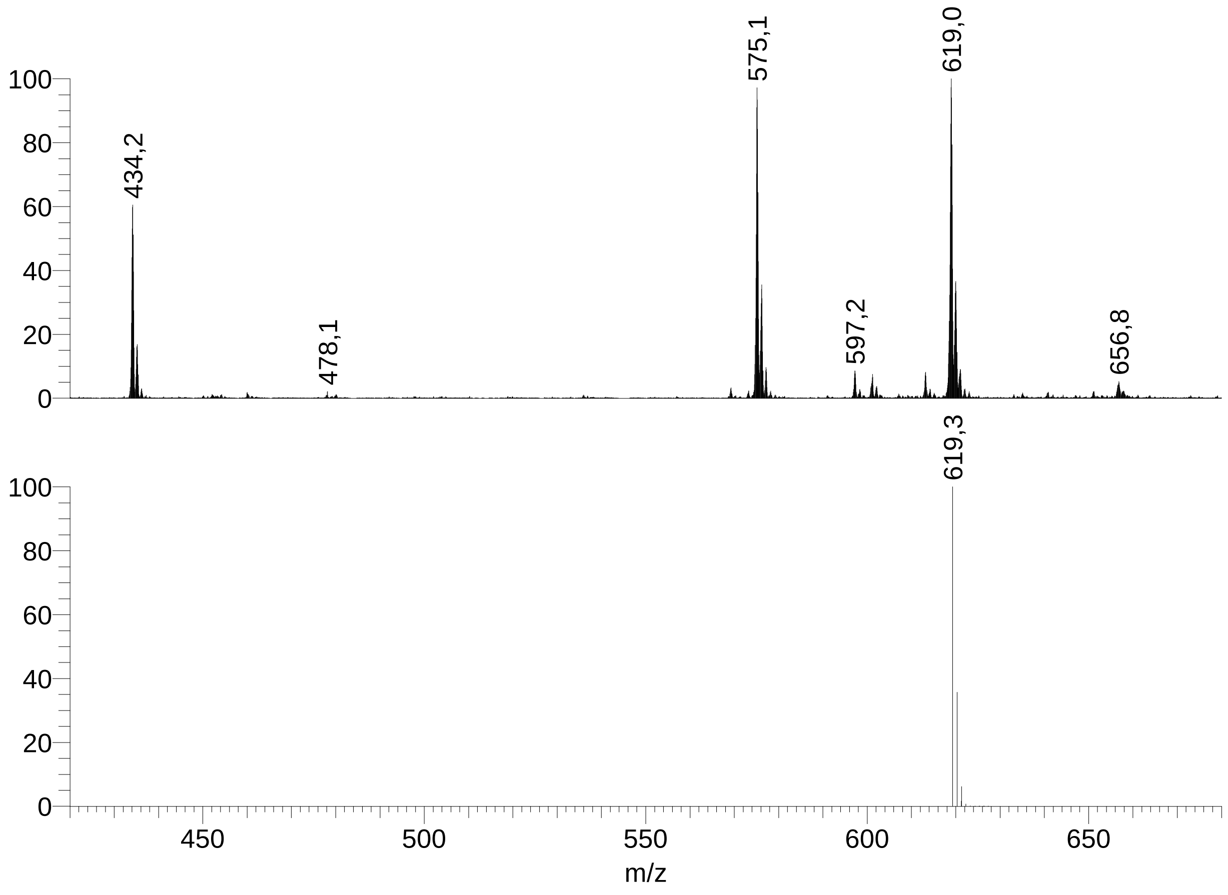

Figure S7. Mass spectral analysis of *At*-DFCC-33 (**1**). Top: experimental positive-ion ESI-mass spectrum; bottom: calculated spectrum for the pseudo-molecular ion $[M+H^+]$.

Isomerization of *At*-DFCC-33 (**1**) and identification of the product as *At*-DNCC-33 (**2**)

Isomerization of *At*-DFCC-33 (**1**)

A sample of the isolated *At*-DFCC-33 (**1**) was dissolved in 2 mL MeOH. An aliquot of 500 µL was diluted with 700 µL of potassium phosphate buffer (100 mM, pH 5) in a UV/Vis-cell, which was kept at room temperature. The isomerisation process was monitored during 280 minutes using an UV/Vis spectrometer (see main text, Figure 3). The isomerization mixture was diluted with 800 µL potassium phosphate buffer and a CD-spectrum was measured. This solution was diluted again with 5 mL potassium phosphate buffer and loaded on a SepPak C18 cartridge, washed with 10 mL water and eluted with 4 mL MeOH. The solvent was removed in vacuum. The sample was dissolved in 50 µL MeOH and diluted with 50 µL potassium phosphate puffer (50 mM, pH 7) for analysis by HPLC and LC-ESI-MS.

Spectral Data of the isomerization product:

UV/Vis (c = 1.2*10^{-5} M). λ_{max} (ε_{rel}) = 286 sh (0.37), 242 sh (1.00).

LC-ESI-MS, m/z (%): 657.0 (9, $[M+K]^+$); 641.0 (5, $[M+Na]^+$); 621.0 (12), 620.0 (37), 619.0 (100, $C_{33}H_{39}N_4O_8$, $[M+H]^+$); 601.2 (21, $[M-H_2O+H]^+$); 575.2 (11, $[M-CO_2+H]^+$); 496.0 (4, [M-C_7H_9NO (ring D)+H]$^+$); 452.2 (15, [M-CO_2-ring D+H]$^+$).

HPLC-analysis of the isomerization product

Authentic *At*-DNCC-33 (**2**) was isolated from a fresh extract of 210 mg of powdered senescent leaves of *A. thaliana* wild type kept in darkness for 7 days. The extract was centrifuged and the clear supernatant was used for analysis by HPLC.

The samples of authentic *At*-DNCC-33 (**2**) and of the product of the isomerization of *At*-DFCC-33 (**1**) were analyzed separately, as well as a 1:1 mixture (see Figure S8).

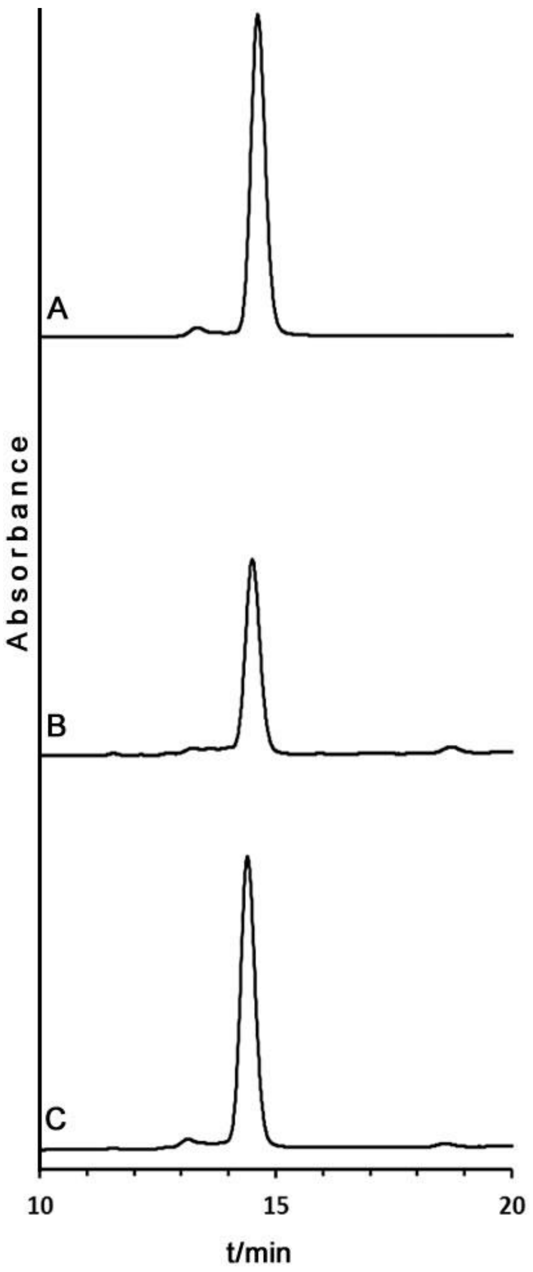

Figure S8. Identification of the product of the isomerization of *At*-DFCC-33 (**1**) with authentic *At*-DNCC-33 (**2**) by HPLC. A) authentic *At*-DNCC-33 (**2**); B) product of the isomerization of *At*-DFCC-33 (**1**); C) 1:1 mixture of both
